# Composition of Rhizosphere Microbial Communities Associated With Healthy and *Verticillium* Wilt Diseased Cotton Plants

**DOI:** 10.3389/fmicb.2021.618169

**Published:** 2021-04-06

**Authors:** Feng Wei, Hongjie Feng, Dezheng Zhang, Zili Feng, Lihong Zhao, Yalin Zhang, Greg Deakin, Jun Peng, Heqin Zhu, Xiangming Xu

**Affiliations:** ^1^State Key Laboratory of Cotton Biology, Institute of Cotton Research of Chinese Academy of Agricultural Sciences, Anyang, China; ^2^State Key Laboratory of Cotton Biology, Zhengzhou Research Base, Zhengzhou University, Zhengzhou, China; ^3^National Institute of Agricultural Botany, East Malling Research, East Malling, United Kingdom

**Keywords:** cotton, *Verticillium dahliae*, rhizosphere soil, amplicon-sequencing, microbial community composition

## Abstract

Rhizosphere microbial communities are known to be related to plant health; using such an association for crop management requires a better understanding of this relationship. We investigated rhizosphere microbiomes associated with *Verticillium* wilt symptoms in two cotton cultivars. Microbial communities were profiled by amplicon sequencing, with the total bacterial and fungal DNA quantified by quantitative polymerase chain reaction based on the respective 16S and internal transcribed spacer primers. Although the level of *V. dahliae* inoculum was higher in the rhizosphere of diseased plants than in the healthy plants, such a difference explained only a small proportion of variation in wilt severities. Compared to healthy plants, the diseased plants had much higher total fungal/bacterial biomass ratio, as represented by quantified total fungal or bacterial DNA. The variability in the fungal/bacterial biomass ratio was much smaller than variability in either fungal or bacterial total biomass among samples within diseased or healthy plants. Diseased plants generally had lower bacterial alpha diversity in their rhizosphere, but such differences in the fungal alpha diversity depended on cultivars. There were large differences in both fungal and bacterial communities between diseased and healthy plants. Many rhizosphere microbial groups differed in their abundance between healthy and diseased plants. There was a decrease in arbuscular mycorrhizal fungi and an increase in several plant pathogen and saprophyte guilds in diseased plants. These findings suggested that *V*. *dahliae* infection of roots led to considerable changes in rhizosphere microbial communities, with large increases in saprophytic fungi and reduction in bacterial community.

## Introduction

Cotton (*Gossypium hirsutum* L.) is an important fiber crop. Cotton *Verticillium* wilt, caused by *Verticillium dahliae*, is one of the most devastating plant diseases worldwide (Klosterman et al., 2009). The pathogen can survive in the soil as resting microsclerotia without a host plant for more than 14 years. In China, its hosts include a number of economically important crops such as potato (*Solanum tuberosum* L.), tomato (*Lycopersicon esculentum* Miller), strawberry (*Fragaria* × *ananassa*), sunflower (*Helianthus annuus*), eggplant (*Solanum melongena* L.), and pepper (*Capsicum annuum* L.). Incidence of cotton wilt increases with increasing densities of *V. dahliae* microsclerotia in soil (Wei et al., 2015). Controlling *Verticillium* wilt is difficult because of the inaccessibility of the pathogen during infection, long-term survival of microsclerotia in soil, and its broad host range (Klosterman et al., 2009). There has been limited success in planting resistant cultivars of upland cotton against wilt in heavy infested fields (Zhang et al., 2012b). In Xinjiang, the main cotton production region in China, crop rotation with non-hosts of *V*. *dahliae* has not been adopted because of the difficulties in changing cropping systems and saline–alkali soils. Soil fumigation with methyl bromide was very effective against *V. dahliae* but has already been banned under the Montreal Protocol (Martin, 2003). Although several remaining fumigants, such as chloropicrin and dazomet, can be used to manage wilt, farmers in China are reluctant to use them in cotton production because of their limited economic benefits.

Plants harbor diverse microbiota both inside and outside their tissues (Vandenkoornhuyse et al., 2015). Rhizosphere microbiota, which closely interact with plant roots, are important for plant health (Berendsen et al., 2012) and crop yield potential (Xu et al., 2015) and influenced by many factors such as plant species and developmental stage, soil properties, nutrient status, land use, and climatic conditions. Selective recruitment of specific microbes by plant roots has been observed (Peiffer et al., 2013; Bai et al., 2015; Zarraonaindia et al., 2015). Suppression of soilborne disease has long been considered as one of the key benefits associated with beneficial microbes in soil (Mendes et al., 2011; Bai et al., 2015; Finkel et al., 2017; Xiong et al., 2017). Microbial diversity and composition are related to plant disease resistance (Wei et al., 2019); high microbial diversity provides greater protection against soilborne pathogens (van Elsas et al., 2002; Mallon et al., 2015). Understanding the association of plant health with rhizosphere microbiota may provide a basis for manipulating soil microbiomes directly (e.g., amending soil with specific microbes) and/or indirectly (e.g., altering management practice) to promote plant health.

Biocontrol of soilborne pathogens has long been a goal of sustainable agriculture, but because of the complexity of the soil environment and resident microbial communities, there are limited numbers of commercial biocontrol products against soilborne diseases in commercial agriculture (Mazzola and Freilich, 2017). To ensure that introduced microorganisms remain effective against pathogens over time in the soil environment, a clear understanding of how the introduced microbes interact with soilborne pathogens and other soil microorganisms is necessary. This knowledge may assist in development of cultural measures to increase the suppressiveness of soil microbiomes against soilborne pathogens and to improve survival (and hence enhance efficacy) of introduced biocontrol microbes.

Multinutrient interactions among resident microbes may be disturbed by plant pathogens, which could cause community reorganization and lead to large-scale collapse and serious degradation of soil ecosystems (van der Putten et al., 2007). There is, however, limited knowledge on the changes in rhizosphere microbiota due to infection of plant roots by pathogens. Recently, several studies have shown that soilborne pathogens can significantly affect soil bacterial composition under field conditions (Shanmugam et al., 2011; Zhang et al., 2011; Wu et al., 2015) or in greenhouse (Mendes et al., 2011; Li et al., 2014). Specific fungal groups, e.g., *Mortierella* spp., may play an important role in the development of soil suppressiveness against *Fusarium* wilt disease in vanilla (*Vanilla planifolia*) (Xiong et al., 2017). However, bacterial and fungal communities are rarely investigated together to understand the nature of disease suppressive soil.

The present study focuses on the changes in rhizosphere microbiome associated with the occurrence of *Verticillium* wilt on two cotton cultivars. Specifically, we quantified *V*. *dahliae* inoculum in rhizosphere soil and assessed the wilt severity for a number of pairs of plants (healthy and diseased plants) in two cultivars. Then we used amplicon metabarcoding to profile rhizosphere microbiome of these paired healthy–wilted plants and quantified the total biomass of fungi and bacteria DNA using quantitative polymerase chain reaction (qPCR) with generic internal transcribed spacer (ITS) and 16S primers. Finally, we established the differences in microbial communities in the rhizosphere between healthy and diseased plants.

## Materials and Methods

### Site Description and Sample Collection

Two commercial monoculture cotton fields (~300 m apart) with the incidence of plants with *Verticillium* wilt >50% were used for sampling in August 2018 in Anyang, Henan Province, China. The soil at the two sites is classified as cambisol type soil (FAO, 1998). At one site (36°03′44″ N, 114°28′52″ E) cv. “Zhongzhimian2” [ZHM2] was grown and cv. “Lumianyan28” [LM28] at the other site (36°03′36″ N, 114°29′04″ E). Both fields were cultivated by farmers using standard cultural practices. Thirty random pairs of neighboring healthy and diseased plants were selected (120 samples in total, 60 plants [30 pairs] per cultivar) for sampling rhizosphere soil on September 3, 2018 (at the boll-forming stage of cotton plants). Each diseased plant was assessed for wilt on an ordinal scale of 0 to 4 as described previously (Wei et al., 2019). For cv. ZHM2, there were 30, 6, 16, and 8 sampled plants with severity score of 0, 1, 2, and 3, respectively; for cv. LM28, there were 30, 8, 16, and 6 sampled plants with wilt severity score of 0, 1, 2, and 3, respectively. Rhizosphere soil samples were collected and stored as described previously (Wei et al., 2019). We first removed the top soil and then dug out of the roots along the base of plants with a soil sampler to maintain the root system integrity as much as possible. Roots were first shaken to remove loosely adhering soil particles; then, each root sample was placed in a sterile plastic bag, kept on ice, and transported to the laboratory within 4 h of sampling the soil. Fine roots were cut into pieces of ~2-cm length with a pair of sterile scissors. Rhizosphere samples were harvested in aliquots of 20-g roots in 1:50 TE buffer by shaking, filtering, and centrifuging. At the same time, soil samples (~100 g) were collected from the position of each sampled plant to estimate the density of *V*. *dahliae* inoculum based on a wet sieving and plating method (Wei et al., 2016).

### DNA Extraction and qPCR

DNA extraction of rhizosphere samples (250 mg) was performed using the MoBio PowerSoil DNA Isolation Kit (MoBio Laboratories, Carlsbad, CA, USA) following published procedures (Wei et al., 2019). Rhizosphere samples (250 mg) were resuspended in 500 μL MoBio PowerSoil bead solution, and DNA was extracted according to the manufacturer's instructions. The extracts were checked on a 1% agarose gel, and the DNA concentration was estimated with a NanoDrop ND-2000 spectrophotometer (NanoDrop Technologies, Wilmington, DE, USA). DNA was stored at −80°C until further analysis.

qPCR of the 16S rRNA and ITS rRNA genes was performed for each sample in triplicate to estimate the total bacterial and fungal abundances (to represent biomass) with a LightCycler 480 system (Roche Diagnostics, Mannheim, Germany). Primer sets F515/R806 (Caporaso et al., 2011) and ITS1f/5.8s (Fierer et al., 2005) were used to quantify bacteria and fungi, respectively. The reactions were conducted in a 20-μL mixture containing 10 μL of SYBR® Premix Ex Taq™ (Tli RNaseH Plus; Takara, China), 0.2 μL of each primer (10 μmol L^−1^), 1 μL of DNA, and 8.6 μL of ultrapure water. Plasmids containing either the 16S rRNA gene fragment or ITS gene fragment were constructed to prepare the respective standard curves, and the plasmid copy numbers were automatically calculated using an online calculator (http://cels.uri.edu/gsc/cndna.html). PCR conditions were as follows: (i) for the bacterial 16S rRNA gene: 95°C for 30 s; 95°C for 30 s, 55°C for 30 s, and 72°C for 30 s for 40 total cycles; and (ii) for the fungal ITS rRNA gene: 95°C for 30 s; 95°C for 30 s, 53°C for 30 s, and 72°C for 30 s for 40 total cycles. No-template controls as well as positive controls with known cycle threshold (Ct) values were included in every qPCR reaction.

### Amplicon Sequencing of Rhizosphere Samples

For bacteria, the V3–V4 hypervariable region of the 16S rRNA gene was amplified in triplicate for each sample using the 341F/805R primers (Herlemann et al., 2011). For fungi, primers ITS5/ITS2 (White et al., 1990) were used to amplify the ITS1 region in triplicates for each sample. PCR reactions and subsequent extraction and purification of amplicons were performed according to the method we used previously (Wei et al., 2019). Sequencing libraries were generated with the Ion Plus Fragment Library Kit 48 rxns (Thermo Scientific, USA) following the manufacturer's recommendations. The quality of each library was assessed on a Qubit 2.0 Fluorometer (Life Technologies, USA). Finally, the libraries were sequenced on an Ion S5™ XL platform (Thermo Fisher Scientific, Waltham, MA) to generate single-end reads. In total, 240 libraries were sequenced: 120 samples (2 cultivars × 30 pairs of healthy and diseased plants) each for 16S rRNA gene and ITS rRNA gene.

### Processing and Analysis of the Sequencing Data

Sequences were processed and filtered separately for 16S and ITS data to retain high-quality sequences following the general pipeline we used previously (Wei et al., 2019) to generate operational taxonomic units (OTUs). These high-quality sequences were first dereplicated, and only those unique reads with at least 2 copies were used in cluster analysis to generate OTUs at 97% sequence identity together with a representative sequence for each OTU. Clustered reads were checked for chimeras using the UPARSE pipeline. An OTU count table was then generated as described previously (Wei et al., 2019). All OTU processing was carried out with the UPARSE pipeline (version 10.0) (Edgar, 2013) unless specified otherwise.

The UTAX algorithm (https://www.drive5.com/usearch/manual9/utax_algo.html) was used to assign each ITS OTU and 16S rRNA OTU representative sequence to taxonomic ranks by alignment with the gene sequences against Unite V7 fungal database (Koljalg et al., 2013) and RDP training set 15 bacterial database (Cole et al., 2014), respectively.

### Statistical Analysis of Sequence Data

The differences in the level of inoculum (microsclerotia) between different levels of disease severities, cultivars, and within-field locations were assessed via analysis of variance (ANOVA) in which inoculum density was logarithmically transformed. Ordinal regression was used to establish whether wilt severity scores are related to cultivars, locations (represented by the pairs of healthy and diseased plants), the level of inoculum, and microbial (bacterial and fungal) biomass (represented by qPCR data). The ordinal package for R version 3.5.4 was used to carry out the ordinal regression analysis.

Before statistical analysis of sequence data, both bacterial and fungal OTU tables were transformed to abundance data by multiplying the relative abundance (sequence reads) of each OTU for each sample with the number of total bacterial 16S rRNA or ITS rRNA copies of the respective sample as determined in the qPCR. All subsequent data analyses were based on this transformed (normalized) OTU data set.

General statistical methodology was similar to previous publications (Tilston et al., 2018; Wei et al., 2019). It should be noted that differences between the two cultivars are confounded with the differences between the two fields; hence, cautions should be excised when interpreting cultivar differences. However, the present study focuses on the differences between healthy and wilted plants.

Alpha-diversity indices, including the observed OTUs, Shannon, and Simpson indices, were calculated with the R vegan 2.3-1 package (Dixon, 2003). The rank of alpha-diversity indices was subjected to ANOVA to assess the differences between healthy and diseased plants with statistical significance derived from a permutation test. Beta-diversity (Bray–Curtis metric) indices were calculated and subjected to non-dimensional scaling analysis as implemented in the vegan package. The effects of cultivar, location within a field (namely, among pairs of plants), and disease status on the beta-diversity indices were assessed with permutational multivariate ANOVA using distance matrices (via the “Adonis” function as implement in the R vegan package version 2.5-7). Similarly, the effects of these experimental factors on the first three principle components were determined via ANOVA.

Further analysis was carried out to identify specific microbial OTUs that differed significantly in their abundances between healthy and diseased plants for each cultivar separately, as well as together through DESeq2 (McMurdie and Holmes, 2013). DESeq2 also implements an algorithm for automatic filtering of OTUs before differential abundance analysis using several criteria, including variance in abundance across samples and overall abundance level. The Benjamini–Hochberg adjustment was used with DESeq2 (Benjamin and Aikman, 1995) to correct for the false discovery rate associated with multiple testing. For tree view graphs, OTU abundances were aggregated at each taxonomic rank (at the SINTAX confidence of 0.7), and these aggregated values were tested for differential abundance between diseased and healthy plants with DESeq2 as above. FunGuild (Nguyen et al., 2016) was used to classify those fungal OTUs with differential abundance between diseased and healthy plants into several broad groups and tested for enrichment using a Fisher exact test in which significance was taken at 0.05 and not corrected for multiple testing.

## Results

### Relationship of Wilt Severity With Bacterial and Fungal Abundances

The overall bacterial and fungal abundances in the rhizosphere soil were estimated with the qPCR technique. The coefficient of determination of the standard curve was 0.993 and 0.988 for bacteria and fungi, respectively; the corresponding efficiencies were 100 and 97%. The number of quantified 16S copies ranged from 1.40 × 10^8^ to 7.06 × 10^9^ per sample (0.25-g soil), with the respective mean and median of 1.29 × 10^9^ and 1.08 × 10^9^. The number of quantified ITS copies ranged from 3.18 × 10^7^ to 1.02 × 10^10^ per sample (0.25-g soil), with the respective mean and median of 1.45 × 10^9^ and 5.85 × 10^8^ copies.

**Figure 1:**
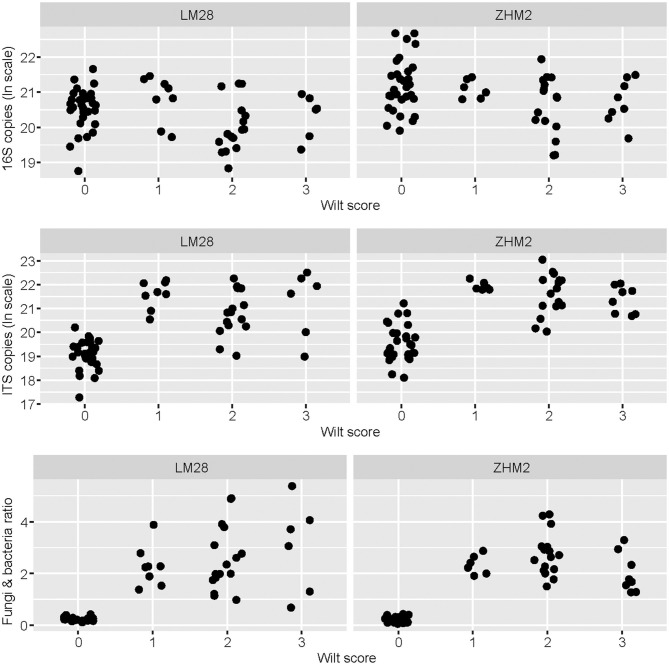
Bacterial 16S and fungal ITS copy number as estimated by qPCR for each rhizosphere soil sample in relation to plant healthy status—healthy plants with the wilt score of zero. Rhizosphere soil samples were collected from neighboring pairs of healthy and diseased (showing *Verticillium* wilt symptoms) cotton plants of two cultivars in two separated fields ~300 m apart.

The density of *V. dahliae* microsclerotia in soil quantified with the wet-sieving method increased with wilt severity (*P* < 0.05) independent of cultivars, but this relationship accounted for only limited variation in the wilt severity (Supplementary Figure 1). None of sampled plants reached a wilt score of 4; no visible root decay was found in all samples. Average colony-forming units (CFU) value was 3.70 per gram of dry soil for healthy plants; average CFU values were 3.26, 4.21, and 5.71 per gram of dry soil for diseased plants with wilt scores of 1, 2, and 3, respectively (Supplementary Figure 1). The ratio between the overall fungal and bacterial abundance also increased (*P* < 0.001) with the increasing wilt score, particularly from healthy to wilt symptoms irrespective of wilt severities (Figure 1). Such a relationship of the wilt severity with the fungal/bacterial biomass ratio was independent of cultivars (Figure 1). This increase in the fungi and bacteria ratio is primarily due to the pronounced increase in the fungal ITS copy number associated with diseased plants (Figure 1). Average ratios of fungal/bacterial abundance were 0.23, 2.31, 2.66, and 2.25 for plants with wilt scores of 0 (healthy) 1, 2, and 3, respectively. There was also less variation in the ratio among healthy samples than among diseased samples (Figure 1) despite the large sample-to-sample variability in both the total bacterial and fungal biomass in the healthy plant samples.

### Overview of the Sequencing Results

There were 4,406 bacterial OTUs, with the top 166 OTUs accounting for 90% of the total reads. The number of sequence reads clustered into OTUs ranged from 27,987 to 72,236 per sample, with the respective mean and median of 49,441 and 47,161. There were 3,856 fungal OTUs, with the top 159 OTUs accounting for 90% of the total reads. Three samples failed the ITS sequencing. Of the remaining 117 samples, the number of sequence reads clustered into OTUs ranged from 31,937 to 71,478 per sample, with the respective mean and median of 64,388 and 68,426. For both ITS and 16S, rarefication curves indicated that sequencing depth is sufficient for all samples (Supplementary Figures 2, 3), except those three sampled that failed ITS sequencing.

Most of the bacterial OTUs could not be assigned to a taxonomic rank below the class with high confidence. At a confidence of 0.8, 85.2, 65.5, 31.9, 21.7, and 15.6% of bacterial OTUs were assigned to the taxonomic rank of phylum, class, order, family, and genus, respectively. The corresponding values for assigning taxonomy to fungal OTUs were 68.8, 37.7, 27.8, 18.9, and 10.8%.

### Alpha Diversity

**Figure 2:**
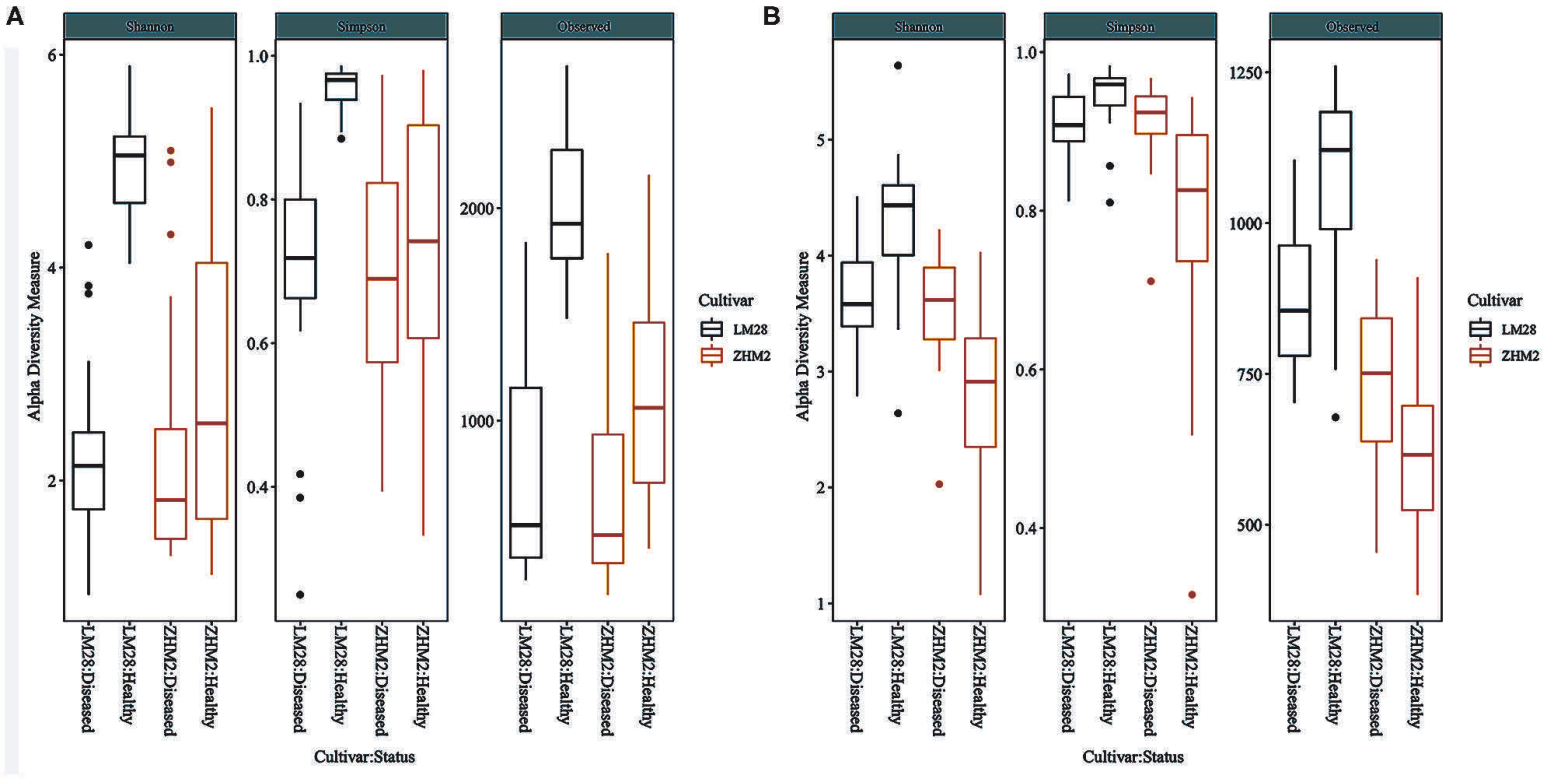
Bacterial **(A)** and fungal **(B)** alpha-diversity indices of rhizosphere soil samples collected from neighboring pairs of healthy and diseased (showing wilt symptoms) cotton plants of two cultivars in two separated fields ~300 m apart. The indices were calculated from 16S and ITS amplicon sequences.

For all three bacterial alpha diversity measures, rhizosphere soil samples from healthy plants had much larger (*P* < 0.001) values than from diseased plants (Figure 2A). Moreover, this difference was even greater on cv. LM28 than cv. ZHM2, as indicated by the interactions (*P* < 0.001) between cultivars and plant disease status. However, fungal alpha-diversity indices showed a different pattern (Figure 2B). LM28 had the same pattern as for bacteria; namely, alpha-diversity indices were greater for healthy plants than for diseased plants. The opposite was true for ZHM2. Such a difference in the response between the two cultivars, i.e., the interaction between cultivar and plant disease status, was highly significant (*P* < 0.001). For both fungi and bacteria, there were no significant differences in the alpha-diversity indices among 30 pairs (i.e., locations within a field) of plant samples.

### Beta Diversity Indices

**Figure 3:**
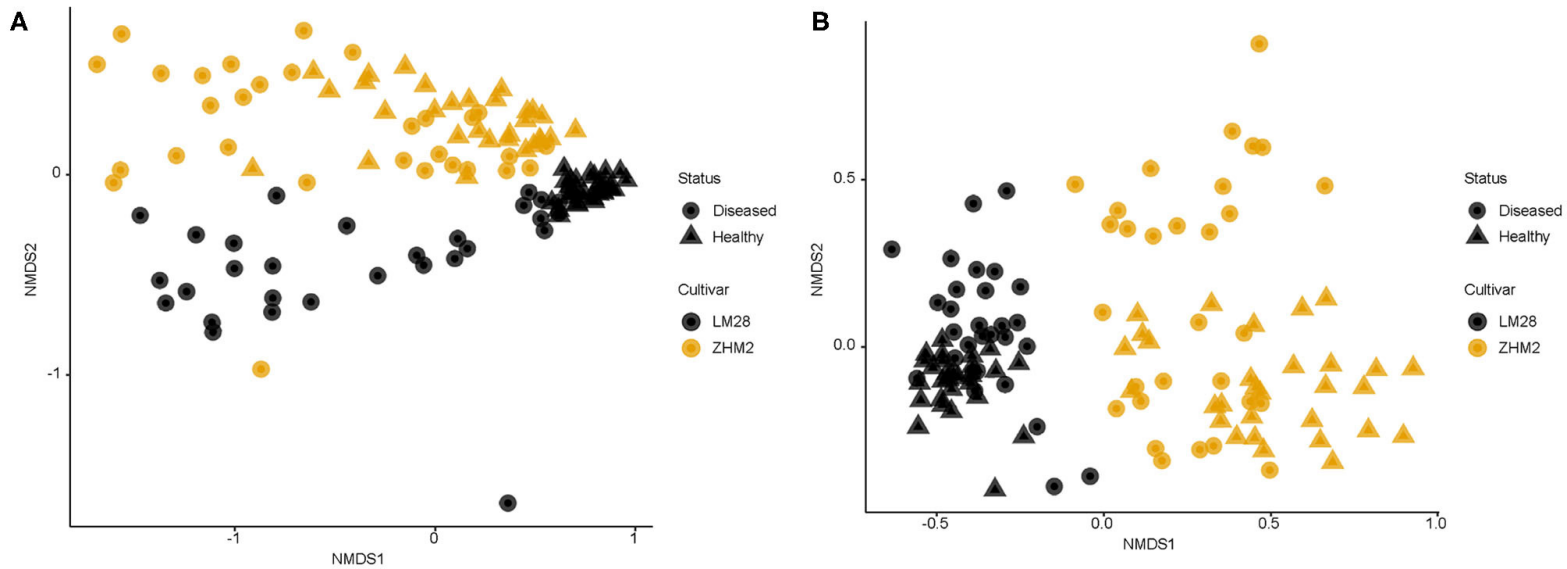
Non-dimensional scaling analysis of bacterial **(A)** and fungal **(B)** community based on Bray–Curtis indices for rhizosphere soil samples collected from neighboring pairs of healthy and diseased (showing wilt symptoms) cotton plants of two cotton cultivars in two separated fields ~300 m apart. 16S and ITS amplicon sequences were used to profile bacterial and fungal communities, respectively.

In contrast to the alpha-diversity indices, beta diversity (Bray–Curtis metric) indices showed consistent differences between diseased and healthy plant samples of both cultivars (Figure 3). In addition, this consistent difference in both fungal and bacterial communities was further supported by the results from principal component analysis (Table 1). Two general patterns can be observed. First, variability among samples from cv. LM28 was less than from cv. ZHM2. Second, variability among diseased samples was far less than among healthy plant samples, particularly for the bacterial community in the rhizosphere of cv. LM28 plants (Figure 3). There were significant differences among locations, namely, between 30 pairs of plants within each cultivar, in the beta-diversity indices as shown by the Adonis analysis.

**Table 1:** Percentage of the total variance in the first three principal components accounted by each term for bacterial and fungal rhizosphere communities of cotton plants.

**Terms**	**Bacteria**			**Fungi**		
	**PC1**	**PC2**	**PC3**	**PC1**	**PC2**	**PC3**
Cultivar [1]	10.4***	12.5***	0.1	20.0***	43.9***	0.4
Plant status [2]	51.5***	2.2***	<0.1	43.3***	9.4***	0.2
Location within cultivar	9.9	1.9	3.8***	19.1	5.1	18.3**
[1] × [2]	10.9***	1.8***	0.1*	0.4	2.7***	0.6*

*Rhizosphere soils from paired of neighboring healthy and diseased plants of two cultivars were sampled and profiled with amplicon sequence for the 16S and ITS regions. Two cultivars were planted in two separate fields ~300 m apart*.

*P < 0.05, **P < 0.01, ***P < 0.001 based on ANOVA.

For both bacterial and fungal communities, the main effects of cultivar and plant wilt status, as well as their interaction on beta-diversity indices, were highly significant (Adonis). For bacteria, cultivar, plant wilt status, and their interactions accounted for 9.1, 16.7, and 6.9% of the total variability, respectively; the corresponding values for the fungal community were 10.3, 18.4, and 4.9%. The interaction was primarily because the difference between the healthy and diseased plant samples was greater for cv. LM28 than cv. ZHM2, especially for the bacterial community.

The first PC accounted for 44.3% of the total variability in the bacterial community, whereas the second and third PCs accounted for only 9.2 and 2.3%, respectively. For the first PC, more than half of its variability among samples was due to the differences between diseased and healthy plant samples (Table 1); cultivar and its interaction with plant wilt status each accounted for ~10% of the total variability. The second PC was primarily affected by cultivars, accounting for 12.5% of the total variability. The third PC was mainly affected by within-field locations (namely, among pairs of plants) but accounting for only 3.8% of the total variability.

For fungal data, the first three PCs accounted for 19.7, 13.3, and 5.4% of the total variability, respectively. The effects of experimental variables on the first three PCs were similar to those for the bacterial community. Thus, the first and second PCs were primarily affected by plant diseased status and cultivar, respectively; nevertheless, the cultivar effect appeared to be more pronounced for the fungal than for the bacterial community (Table 1).

### Comparison of Individual OTUs Between Diseased and Healthy Plant Samples

#### Rhizosphere Bacterial Community Composition

On cv. LM28, 1,543 bacterial OTUs passed the default DESeq2 filtering criteria and were subjected to statistical comparison. Of the 1,543 OTUs, 1,027 differed in their abundance between diseased and healthy plant samples. For 1,007 of the 1,027 OTUs, the abundance was less in the rhizosphere of diseased plants than healthy plants (Supplementary Figure 4A). For cv. ZHM2, only 1,243 bacterial OTUs passed the default DESeq2 filtering criteria. Of the 1,243 OTUs, 640 differed in their abundance between diseased and healthy plant samples. For 631 of the 640 OTUs, the abundance was less in the rhizosphere of diseased plants than healthy plants (Supplementary Figure 4B).

**Figure 4:**
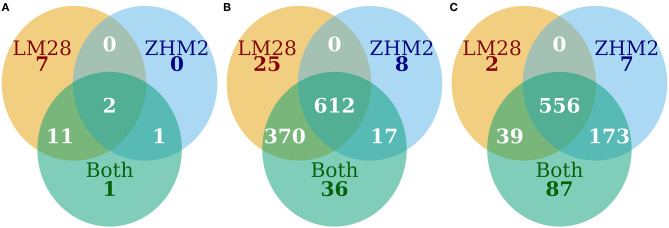
The number of OTUs that differed (*P* ≤ 0.05) in their abundance between diseased and healthy plant samples. **(A)** Bacteria increased in diseased samples, **(B)** bacteria decreased in diseased samples, and **(C)** fungi increased in diseased samples.

When both cultivars were analyzed together, 1,468 bacterial OTUs passed the default DESeq2 filtering criteria. Of the 1,468 OTUs, 1,050 differed in their abundance between diseased and healthy plant samples. For 1,035 of the 1,050 OTUs, the abundance was less in the rhizosphere of diseased plants than healthy plants (Figures 4A,B). The majority of these OTUs with reduced abundance in the diseased plants were shared between the two cultivars with only 25 and 8 unique to LM28 and ZHM2, respectively (Figure 4B). Except for Gammaproteobacteria, the relative abundance of almost all bacterial groups was reduced in the rhizosphere of diseased plants of both cultivars, including several well-known taxonomy groups containing beneficial microbes, such as *Bacilli* (Firmicutes) and Gemmatimonadetes (Supplementary Figure 6).

#### Rhizosphere Fungal Community Composition

On cv. LM28, only 1,142 fungal OTUs passed the default DESeq2 filtering criteria and were subjected to statistical comparison. In total, 597 OTUs differed in their abundance between diseased and healthy plant samples; for all these OTUs, the abundance was greater in the rhizosphere of diseased plants than healthy plants (Supplementary Figure 5A). For cv. ZHM2, only 1,272 fungal OTUs passed the default DESeq2 filtering criteria. Of the 1,272 OTUs, 738 differed in their abundance between diseased and healthy plant samples. For 736 of these 738 OTUs, the abundance was greater in the rhizosphere of diseased plants than healthy plants (Supplementary Figure 5B).

When both cultivars were analyzed together, 1,856 fungal OTUs passed the default DESeq2 filtering criteria. Of the 1,856 OTUs, 855 differed in their abundance between diseased and healthy plant samples. For all these 855 OTUs, the abundance was greater in the rhizosphere of diseased plants than healthy plants.

**Figure 5:**
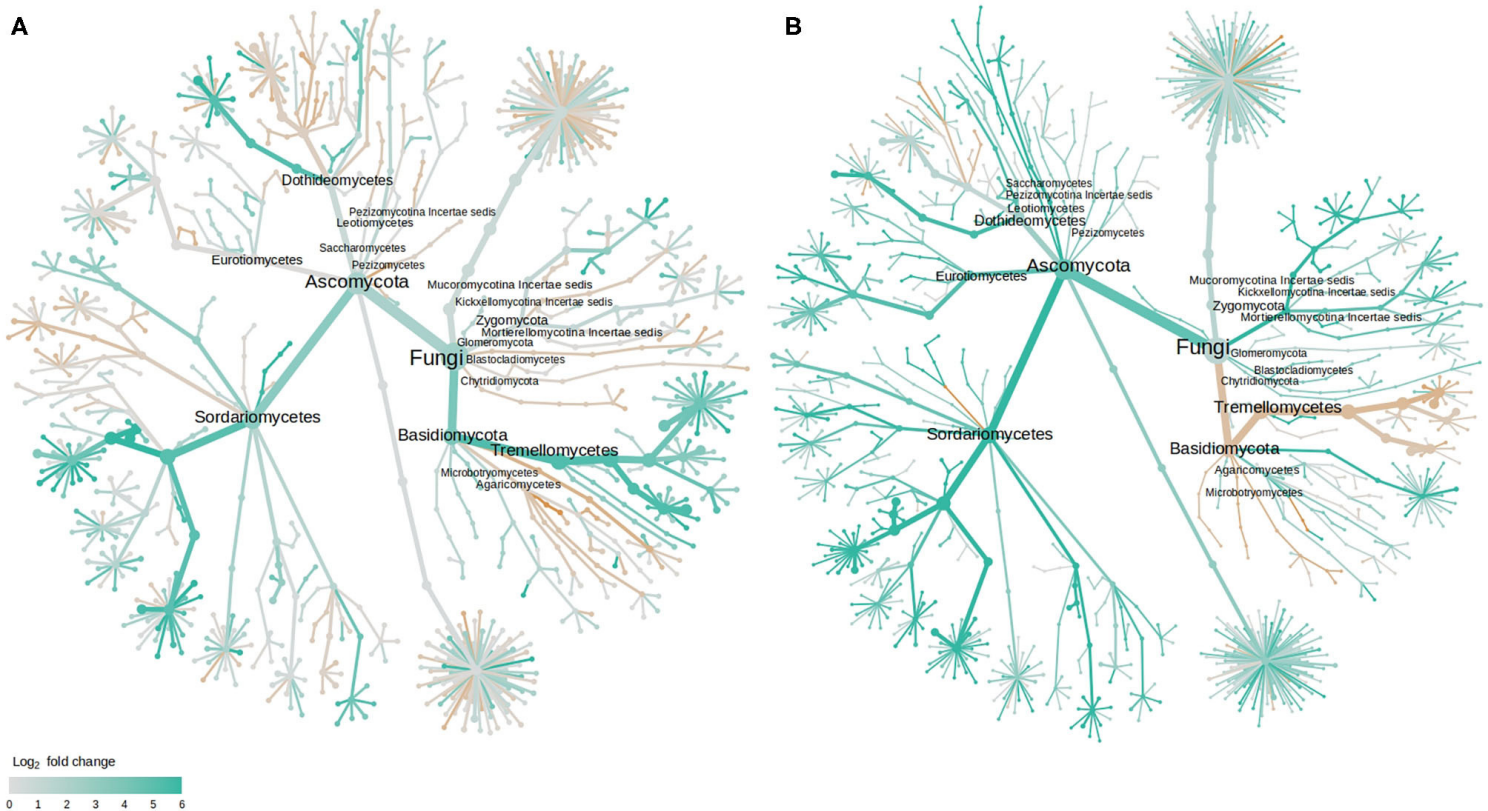
Tree views of the differences in the fungal abundance in the rhizosphere of healthy and diseased cotton plants: **(A)** cv. LM28, and **(B)** cv. ZHM2; the difference in abundance is expressed as log_2_FoldChange, with values >0 indicating that the abundance of specific OTUs was greater in diseased samples than in healthy samples. Label and node represent the abundance of rhizosphere fungi at the class rank.

As with bacteria, the majority of those fungal OTUs with increased abundance in the rhizosphere of diseased plants were shared between the two cultivars with only 2 and 7 unique to LM28 and ZHM2, respectively (Figure 4C). Taxonomy heat trees (Figure 5) were constructed with Metacoder (Foster et al., 2017) for each cultivar to illustrate the differences in fungal abundances between healthy and diseased samples at specific taxonomy ranks. There were clear distinctions in the Tremellomycetes and a clade (Sordariales order) within the Sordariomycetes that had increased abundance in diseased plant samples over the healthy plants in cv. ZHM2 but not in LM28. Similar heat trees for bacterial OTUs showed very little difference (Supplementary Figure 6) between the two cultivars.

The fungal taxonomy was annotated using the online version of FUNGuild (http://www.stbates.org/guilds/app.php). Enrichment analysis (Fisher exact test with uncorrected *P*-values) of trophic level annotations of those OTUs with increased abundance in diseased samples indicated an increase in saprotrophs and a decrease in symbionts (Table 2). Enrichment of “Guilds” indicated a decrease in arbuscular mycorrhizal in cv. LM28 and an increase in several plant pathogen guilds (Table 3).

**Table 2:** Differences in the number of putative fungal functional groups (identified by FUNGuild “trophic level” annotations) in the rhizosphere of healthy and diseased cotton plants in two cultivars (LM28 and ZHM2).

**Annotated**		**LM28**			**ZHM2**			**Both**		
		**Sig**.	**Odds ratio**	***P***	**Sig**.	**Odds ratio**	***P***	**Sig**.	**Odds ratio**	***P***
Unknown	2,190	283	1.38	<0.001	394	1.22	0.004	462	1.21	0.003
Sap^a^	543	156	0.62	<0.001	190	0.63	0.000	199	0.7	<0.001
Path^b^	82	14	1.04	1.000	14	1.29	0.500	19	1.1	0.803
Sym^c^	83	5	2.96	0.010	9	2.03	0.039	12	1.77	0.070
Sap-Path	85	22	0.69	0.133	23	0.81	0.376	29	0.75	0.195
Sap-Sym	72	16	0.80	0.452	21	0.75	0.275	22	0.84	0.440
Path-Sym	26	6	0.77	0.618	6	0.95	0.820	7	0.95	0.830
Sap-Path-Sym	268	95	0.50	<0.001	79	0.75	0.030	105	0.65	0.001

*Individual OTUs with significant differences in its abundance between diseased and healthy plants determined by DESeq2 analysis. Odds ratio of <1 indicates a higher proportion of OTUs in the particular fungal functional group in the microbiome of diseased samples, and P-value indicates whether the odds ratio differs significantly from 1.0*.

*^a^Saprotroph, ^b^Pathotroph, ^c^Symbiotroph*.

**Table 3:** Differences in the number of putative fungal functional groups (identified by FUNGuild “trophic level” annotations) in the rhizosphere of healthy and diseased cotton plants in two cultivars (LM28 and ZHM2).

**Annotated**		**LM28**			**ZHM2**			**Both**		
		**Sig**.	**Odds ratio**	***P***	**Sig**.	**Odds ratio**	***P***	**Sig**.	**Odds ratio**	***P***
Undefined sap	433	143	0.54	<0.001	172	0.55	<0.001	178	0.62	<0.001
Plant path undefined sap	14	8	0.31	0.012	8	0.38	0.045	9	0.40	0.036
Dung sap undefined sap	5	4	0.22	0.035	4	0.27	0.062	4	0.32	0.090
Arbuscular mycorrhizal	71	5	2.52	0.035	9	1.73	0.140	12	1.51	0.215
Fungal parasite Undefined sap	133	52	0.45	<0.001	29	1.01	1.000	53	0.64	0.009
Endophyte fungal parasite plant path	9	5	0.32	0.048	5	0.39	0.152	5	0.46	0.178
Animal path endophyte Fungal parasite Plant path Wood sap	21	10	0.37	0.019	11	0.42	0.034	12	0.45	0.030
Endophyte Litter sap Soil sap undefined sap	39	15	0.46	0.020	19	0.45	0.009	20	0.50	0.014
Animal path Dung sap endophyte Epiphyte Plant sap Wood sap	19	10	0.34	0.008	12	0.35	0.008	12	0.40	0.022

*Individual OTUs with significant differences in its abundance between diseased and healthy plants determined by DESeq2 analysis. Odds ratio of <1 indicates a higher proportion of OTUs in the particular fungal functional group in the microbiome of diseased samples, and P-value indicates whether the odds ratio differs significantly from 1.0. Only guilds with P ≤ 0.05 in any of the contrasts are shown*.

## Discussion

In the present study, similar to our previous finding (Wei et al., 2015), wilt severity increased significantly with the increasing *V*. *dahliae* inoculum density in the rhizosphere. However, the inoculum level explained only a very small proportion of the variability in observed wilt severities among plants, indicating that most differences in wilt severities are likely due to other factors rather than the differing inoculum densities.

Total bacterial biomass, as indicted by qPCR results, was significantly higher in the rhizosphere of healthy plants than that of diseased plants; the opposite was true for the total fungal biomass. Similar results in total bacteria were also observed for potato plants with high and low levels of potato common scab severity (Shi et al., 2019). Suppressive soil against *Fusarium* wilt has higher populations of bacteria than the wilt-conducive soil that has higher populations of fungi (Peng et al., 1999). The occurrence of cotton *Verticillium* wilt appears to be accompanied by increased fungal abundance in the rhizosphere. The ratio of total fungi with total bacteria in the rhizosphere of wilt cotton plants is much > 1; the opposite is true for the healthy plants. Therefore, the ratio of total fungal to total bacterial biomass can be considered as an indicator of cotton *Verticillium* wilt occurrence. This agrees with previous findings that the ratio of fungi to bacteria shows increasing trends in the soil of continuous cropping that leads to severe soilborne disease of *Panax notoginseng* (Dong et al., 2016). Interestingly, this ratio appears to be less variable for healthy plants than for diseased plants, suggesting that healthy plants are associated with stable fungal/bacterial communities.

Microorganisms are one indicator of soil health, particularly disease suppressiveness (Epelde et al., 2014; Ferris and Tuomisto, 2015; van Bruggen et al., 2015). High microbial diversity can improve community stability (Lefcheck et al., 2015; Delgado-Baquerizo et al., 2016). However, the relationship between microbial diversity and disease development varies with studies. For instance, a higher diversity in soil bacteria is associated with healthy plants in *P*. *notoginseng* (Wu et al., 2015) and cotton (Zhang et al., 2011), whereas the opposite was observed in tomato plants (Li et al., 2014). In the present study, higher bacterial alpha-diversity indices were found in healthy plants than in wilt diseased plants, but this is not true for fungal community. Higher diversity in soil bacteria is often associated with greater resistance to pathogens (Garbeva et al., 2004; Mallon et al., 2015). The increase of soil resistance/tolerance to pathogens may be related to the complexity of the interaction network of microorganisms in soil (Shi et al., 2019). Complex microbial community interaction can regulate the stability of the community (Eisenhauer et al., 2013), thus limiting pathogen increase.

Soilborne pathogens can have profound impacts on nutrient availability and plant root exudates in rhizosphere, which, in turn, may affect microbial communities (Cook et al., 1995). The present study showed large and consistent effects of a soilborne vascular disease on both fungal and bacterial communities in the rhizosphere of cotton plants. Rhizosphere microbiomes have been showed to differ between healthy and soilborne diseased plants (Mendes et al., 2011; Shanmugam et al., 2011; Zhang et al., 2011; Wu et al., 2015). However, a recent study showed that bacterial community in the geocaulosphere soil could be distinguished according to potato scab severity, but not in rhizosphere soil (Shi et al., 2019). This difference could be because that common scab of potato mainly invades through tubers, whereas many other soilborne pathogens directly infect root systems. As a vascular pathogen of the cotton, *V. dahliae* infects roots and then colonizes vascular tissues, causing plant wilting, but usually does not cause root decay. However, fungal colonization in vascular tissues is expected to result in considerable changes in plant physiology and hence in root exudates. We thus speculate that it is the change in root exudates that may largely be responsible for the resulting differences in rhizosphere microbiomes between healthy and diseased plants.

In the present study, the differences in the rhizosphere microbiome between diseased and healthy plants within the same cultivars are much greater than differences between cultivars. Cultivar differences may be exaggerated as the two cultivars were grown separately in two neighboring fields and thus are confounded with the differences in microbial communities between the two fields, which is well-known (Edwards et al., 2015). Such a spatial effect was also illustrated by the significant differences between pairs of plants (representing different locations within a field). Precisely for this reason, we sampled scheme neighboring diseased and healthy plants of the same cultivar to minimize the compounding effects of disease phenotype and microbial variability in space. Thus, the differences in the rhizosphere microbial community between diseased and healthy plant within the pairs of plants are more likely to be directly related to the wilt development. The important question, however, remains whether the large community differences in the rhizosphere between diseased and healthy plants are a consequence of the infection by *V*. *dahliae* and/or subsequent wilt development that has affected root exudate composition leading to changes in rhizosphere microbiome.

Differences in abundance of individual OTUs between the diseased and healthy plants have a consistent pattern: nearly all those bacterial OTUs with differential abundance had much higher abundance in healthy plants than in diseased plants, and the opposite was true for the fungal OTUs. Many bacterial and some fungal groups have been shown to associate with cotton plant tolerance to development of wilt caused by *V*. *dahliae* (Wei et al., 2019). Many specific bacterial OTUs have higher abundance in rhizosphere of healthy plants than diseased plants. For instance, the abundance of Acidobacteria is reduced in rhizosphere of diseased plants, agreeing with a recent finding that the decrease of this phylum is linked to wilt development in olive, also caused by *V. dahliae* (Fernández-González et al., 2020). Furthermore, the abundance of several well-known taxonomy groups containing beneficial microbes, such as *Bacilli* (Firmicutes) and Gemmatimonadetes, was reduced in the rhizosphere of diseased plants, consistent with previous findings (Wei et al., 2019; Fernández-González et al., 2020). In contrast, the abundance of Gammaproteobacteria was increased in diseased plants in both cultivars. Gammaproteobacterial diversity and community members have been identified as potential health indicators (Köberl et al., 2017). For example, healthy banana (*Musa acuminata* L.) plants have increased presence of potentially plant-beneficial *Pseudomonas* and *Stenotrophomonas*, whereas diseased plants had a high level of Enterobacteriaceae known for their plant degradation ability (Köberl et al., 2017). Decreased abundance associated with diseased plants was also observed for arbuscular mycorrhizal fungi (AMF). AMF colonization may have increased the expression of pathogenesis-related genes and lignin synthesis-related genes more strongly, thus leading to induced resistance against *V*. *dahliae* in cotton (Zhang et al., 2018). In addition, AMF can also induce changes in the composition of cotton root exudates, contributing to bioactive effects against germination of *V*. *dahliae* conidia (Zhang et al., 2012a). Similarly, increased abundance of fungal pathogens in general together with reduced abundance of beneficial microbes may explain yield (mainly due to soilborne diseases) declining potential observed in continuous cotton monocropping systems (Wei and Yu, 2018).

The present study showed that a much greater proportion of rhizosphere microbial OTUs differed in their abundance between neighboring diseased and healthy plants of the same cultivars than between cultivars with differing susceptibility to *V*. *dahliae*. This may suggest that much of these differences between diseased and healthy plants may have resulted from postinfection or postdisease development as consequences of changes in root exudates and/or volatiles from plants associated with pathogen infection and subsequent disease development. Thus, rhizosphere of diseased plants has greater abundance of fungal saprophytes than healthy plants.

## Conclusion

This study demonstrated that *V*. *dahliae* infection and subsequent disease development can lead to large changes in rhizosphere microbiomes in cotton. In addition to high *V*. *dahliae* inoculum density, a high fungal/bacterial biomass ratio in rhizosphere is an indicator of wilt disease development. Much of the increased fungal abundance in diseased roots is likely contributable to increased fungal saprophytes.

## Data Availability Statement

Raw sequence data reported in this paper have been deposited to the European Nucleotide Archive (ENA) under accession number PRJEB39152.

## Author Contributions

FW, XX, JP, and HZ planned, designed the research, and experiments. FW, HF, DZ, ZF, LZ, and YZ performed the experiments. FW, XX, and GD analyzed the data. FW and XX wrote the manuscript. FW acquired the funds for the study. All authors read and approved the final manuscript.

## Conflict of Interest

The authors declare that the research was conducted in the absence of any commercial or financial relationships that could be construed as a potential conflict of interest.

## Supplementary Material

Supplementary Figure 1 Estimated *Verticillium dahliae* inoculum densities (CFU/g) in soil plotted against wilt scores of individual cotton plants.

Supplementary Figure 2 Five examples of rarefaction curves for bacteria. All curves terminated at the number of sequences obtained in the examples.

Supplementary Figure 3 Five examples of rarefaction curves for fungi. All curves terminated at the number of sequences obtained in the examples.

Supplementary Figure 4 Plots of DeSeq2 analysis results for bacterial OTUs with significant (*P* < 0.05) differences between the healthy and diseased plants for **(A)** cv. LM28, **(B)** cv. ZHM2; mean expression is the average number of sequence reads for each OTU, and log_2_FoldChange is the log_2_ of the ratio in the number of sequence reads between the healthy and diseased plants. Positive log_2_FoldChange indicates that the relative abundance of specific OTUs was greater in the healthy plant samples than in diseased plant samples.

Supplementary Figure 5 Plots of DeSeq2 analysis results for fungal OTUs with significant (*P* < 0.05) differences between the healthy and diseased plants for **(A)** cv. LM28, **(B)** cv. ZHM2; mean expression is the average number of sequence reads for each OTU and log_2_FoldChange is the log_2_ of the ratio in the number of sequence reads between the healthy and diseased plants. Positive log_2_FoldChange indicates that the abundance of specific OTUs was greater in the healthy plant samples than in diseased plant samples.

Supplementary Figure 6 Tree views of the differences in bacterial abundance in the rhizosphere of healthy and diseased cotton plants: **(A)** cv. LM28, and **(B)** cv. ZHM2; the difference in abundance is expressed as log_2_FoldChange with values >0 indicating that the abundance of specific OTUs was greater in diseased samples than in healthy samples. Label and node represent the abundance of rhizosphere fungi at the class rank.
