# Supplementary figures and images for: Composition of Rhizosphere Microbial Communities Associated With Healthy and Verticillium Wilt Diseased Cotton Plants
Source: Front Microbiol. 2021 Apr 6;12:618169. doi: 10.3389/fmicb.2021.618169 (PMC8057349; doi:10.3389/fmicb.2021.618169)

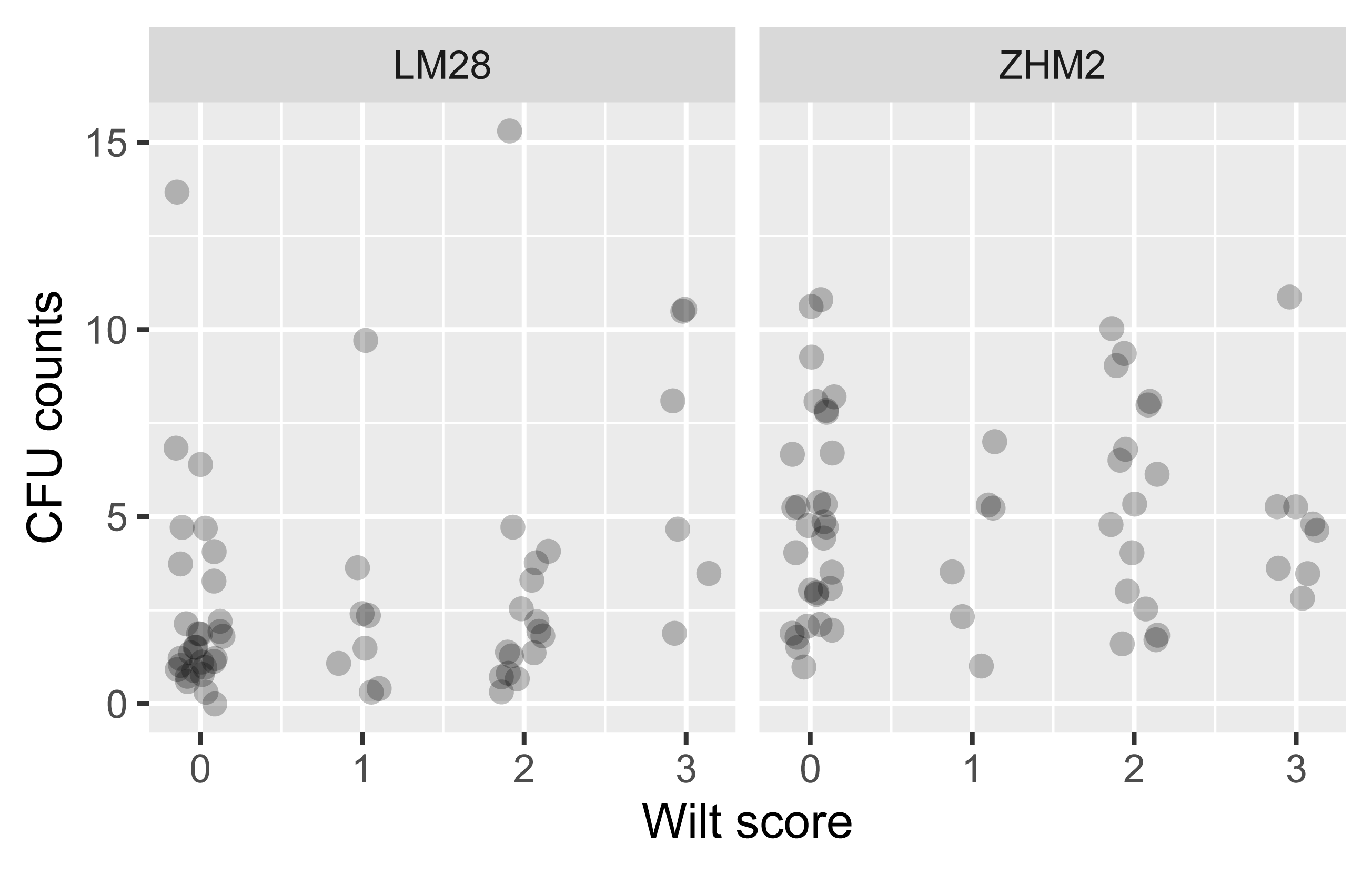

Supplement: Supplementary Figure 1 — Estimated Verticillium dahliae inoculum densities (CFU/g) in soil plotted against wilt scores of individual cotton plants. [file Image_1.TIF]

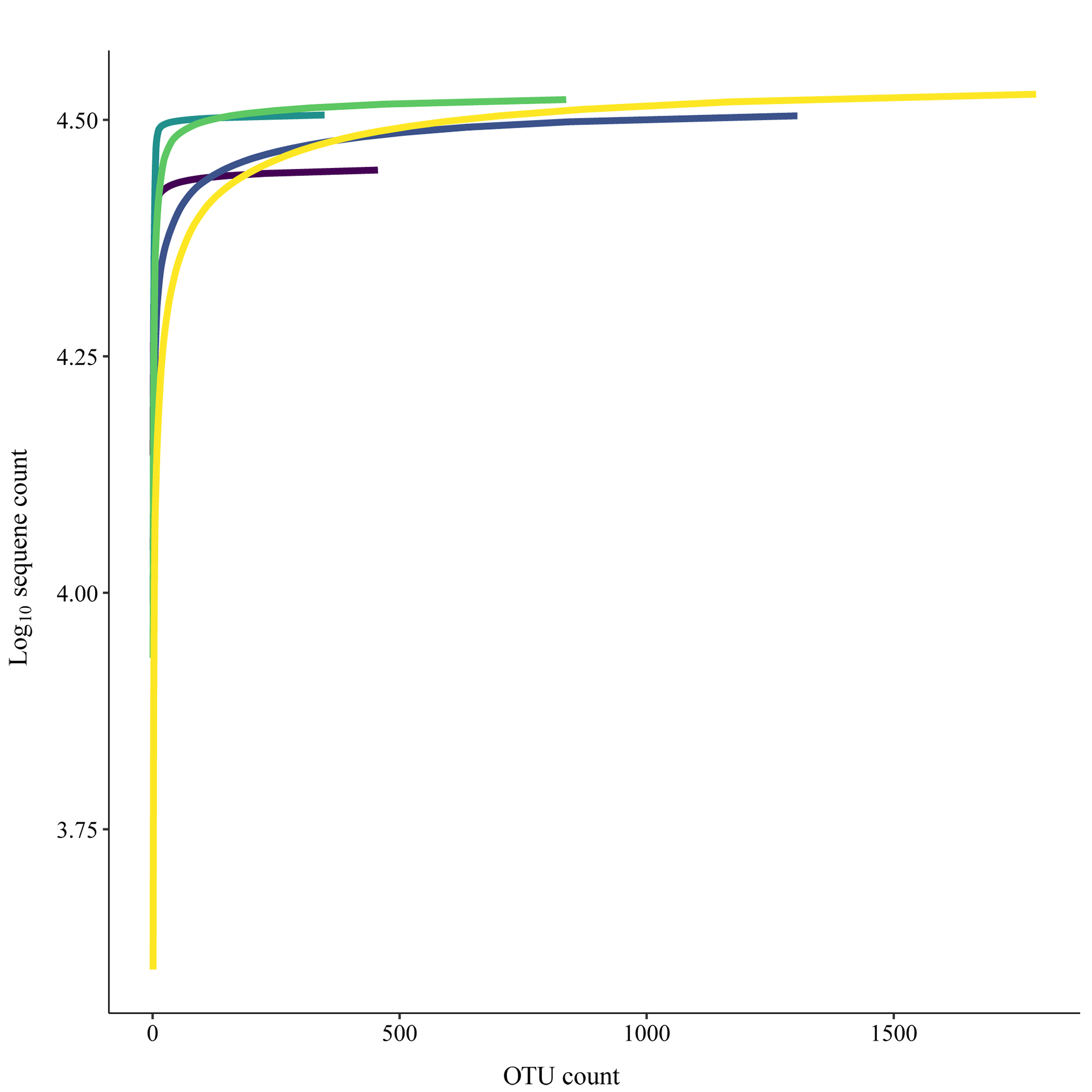

Supplement: Supplementary Figure 2 — Five examples of rarefaction curves for bacteria. All curves terminated at the number of sequences obtained in the examples. [file Image_2.TIF]

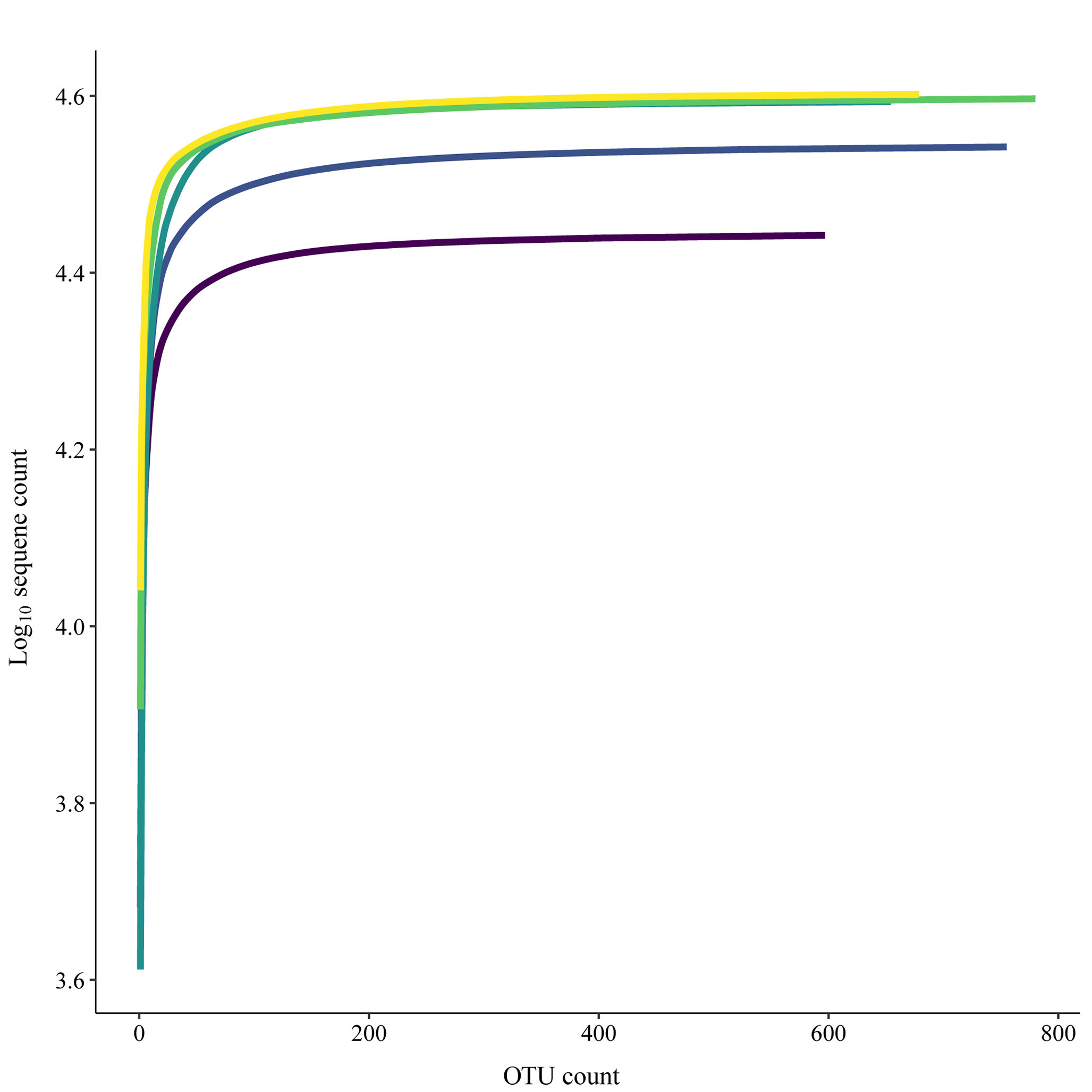

Supplement: Supplementary Figure 3 — Five examples of rarefaction curves for fungi. All curves terminated at the number of sequences obtained in the examples. [file Image_3.TIF]

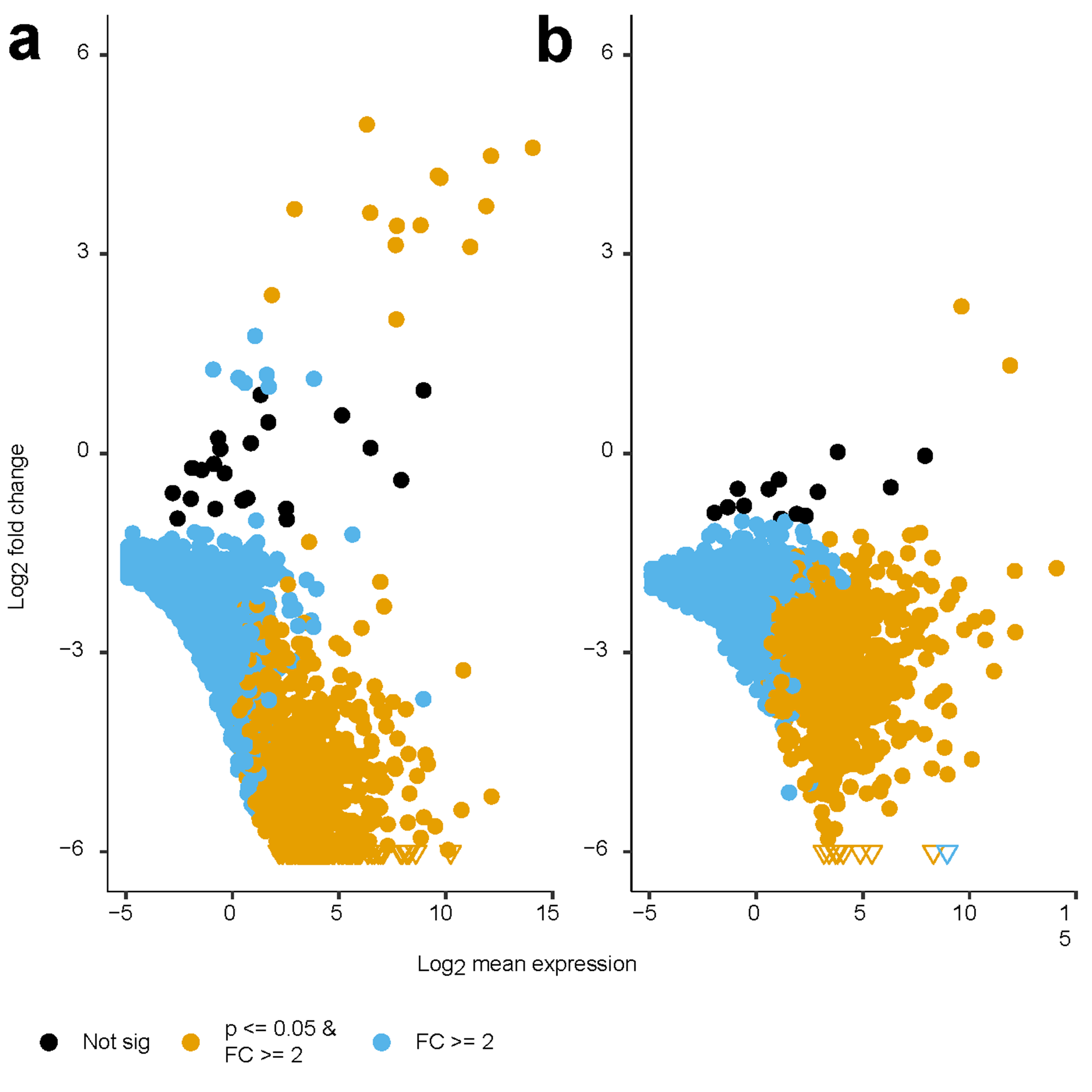

Supplement: Supplementary Figure 4 — Plots of DeSeq2 analysis results for bacterial OTUs with significant (P < 0.05) differences between the healthy and diseased plants for (A) cv. LM28, (B) cv. ZHM2; mean expression is the average number of sequence reads for each OTU, and log2FoldChange is the log2 of the ratio in the number of sequence reads between the healthy and diseased plants. Positive log2FoldChange indicates that the relative abundance of specific OTUs was greater in the healthy plant samples than in diseased plant samples. [file Image_4.TIF]

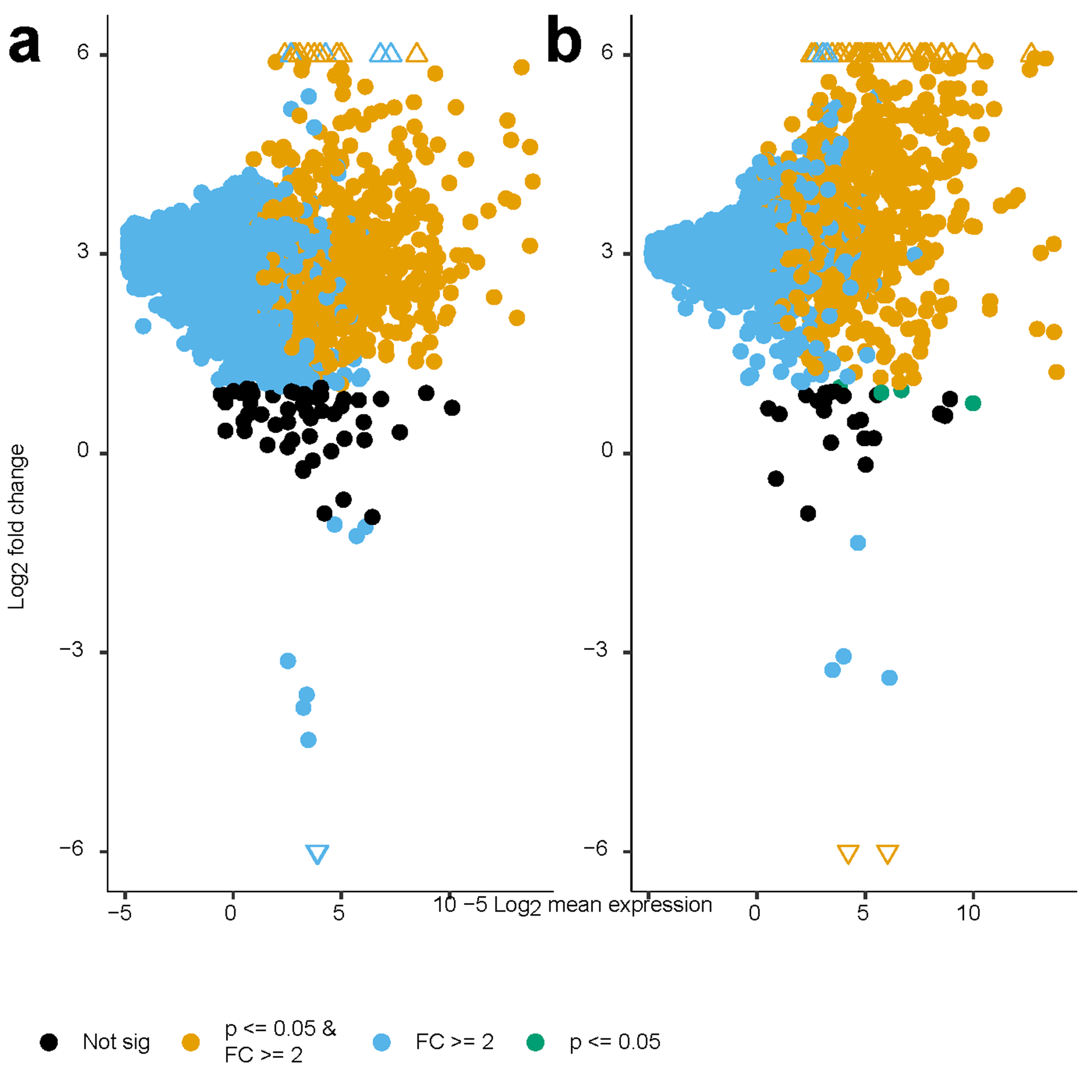

Supplement: Supplementary Figure 5 — Plots of DeSeq2 analysis results for fungal OTUs with significant (P < 0.05) differences between the healthy and diseased plants for (A) cv. LM28, (B) cv. ZHM2; mean expression is the average number of sequence reads for each OTU and log2FoldChange is the log2 of the ratio in the number of sequence reads between the healthy and diseased plants. Positive log2FoldChange indicates that the abundance of specific OTUs was greater in the healthy plant samples than in diseased plant samples. [file Image_5.TIF]

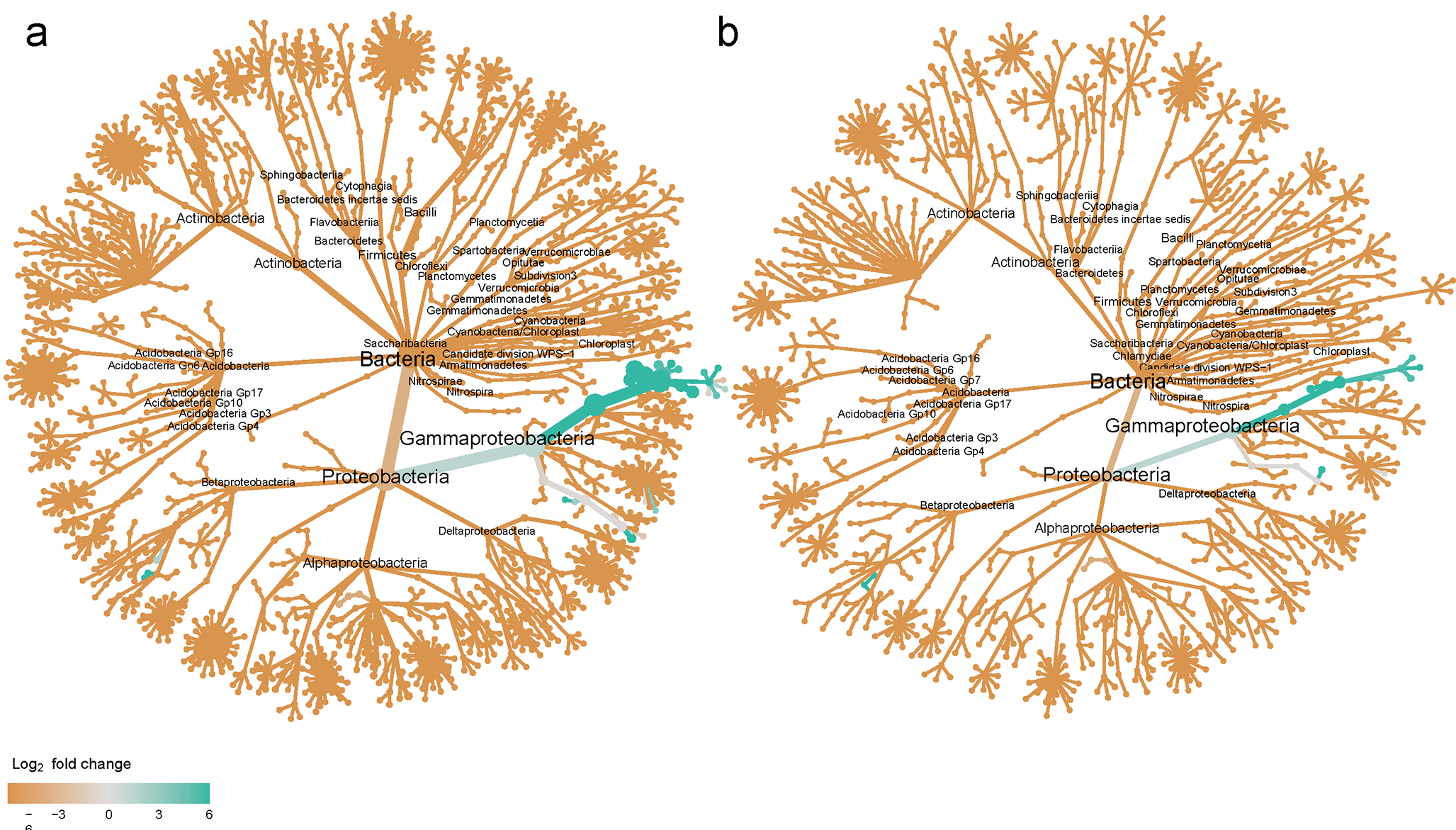

Supplement: Supplementary Figure 6 — Tree views of the differences in bacterial abundance in the rhizosphere of healthy and diseased cotton plants: (A) cv. LM28, and (B) cv. ZHM2; the difference in abundance is expressed as log2FoldChange with values >0 indicating that the abundance of specific OTUs was greater in diseased samples than in healthy samples. Label and node represent the abundance of rhizosphere fungi at the class rank. [file Image_6.TIF]
